# Pea protein isolate characteristics modulate functional properties of pea protein–cranberry polyphenol particles

**DOI:** 10.1002/fsn3.2335

**Published:** 2021-05-24

**Authors:** Renee Cilliers Strauch, Mary Ann Lila

**Affiliations:** ^1^ Plants for Human Health Institute North Carolina State University Kannapolis NC USA; ^2^ Department of Food, Bioprocessing and Nutrition Sciences North Carolina State University Kannapolis NC USA

**Keywords:** by‐products, cranberry polyphenols, digestion, pea protein isolate, solubility

## Abstract

Plant polyphenols have a natural binding affinity for proteins, and their interaction can be exploited to form diverse aggregate particles. Protein–polyphenol particles utilized as food ingredients allow consumers to incorporate more health‐benefiting plant bioactives into their diets. The functional properties of the protein–polyphenol particles can be influenced by many factors, including complexation conditions and starting material properties. Here, cranberry polyphenols extracted from pomace were complexed with nine pea protein isolate starting materials with different physical (particle size and protein content) and chemical (hydrolyzed and oxidized) properties to investigate the impact of protein characteristics on particle functionality. Chemical differences between proteins affected polyphenol binding; oxidized protein isolate (specifically, VegOtein N) bound 12%–27% more polyphenols than other isolates. Polyphenol binding to proteins decreased digestion rates in vitro, averaging 25% slower gastric (pepsin) digestion and a 35% slower intestinal (pancreatin) digestion. Physical differences in protein starting materials affected digestibility; isolate with the largest particle size (specifically, Nutralys F85G) produced particles with the lowest digestion rate. Solubility was impacted by both the process of forming particles and by polyphenol binding; control particles were 56% less soluble, and protein–polyphenol particles up to 75% less soluble, than unmodified proteins. The solubility of unmodified protein isolate starting materials varied widely according to the manufacturing process, but, after complexation, protein–polyphenol particles produced from all protein sources exhibited a similar depressed level of solubility. The desired functional properties of the protein–polyphenol particle food ingredients will be considerably influenced by the properties of the protein isolate starting material.

## Graphical abstract

Plant polyphenols have a strong binding affinity for proteins and their interaction can be exploited to make protein–polyphenol particle food ingredients that stabilize high levels of health‐benefiting plant bioactives. We investigated how the functional properties of these particles, including polyphenol binding, protein digestion, and solubility, which are vital to the color, sensory, and nutritive quality of resulting food products, are dependent on the chemical and physical characteristics of the protein starting material.

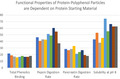

## 1 INTRODUCTION

The manifold health benefits of a diet rich in polyphenols, such as the proanthocyanidins, anthocyanins, and other flavonoids found in cranberries, have been demonstrated by numerous epidemiological and intervention studies (Anhe et al., 2015; Neto, 2007; Rodriguez‐Mateos et al., 2016). Multiple daily servings of fruits and vegetables are recommended by dietary guidelines, and consumers are increasingly interested in consuming these health‐promoting compounds. However, there are some real and perceived disadvantages preventing consumers from incorporating adequate quantities of fresh fruits and vegetables into their diet, including the expense, difficulty in storing and preparing, as well as seasonal accessibility. Additionally, bioactive polyphenolics are rapidly degraded upon exposure to light, heat, and oxygen resulting in short shelf life and limited processability. Innovative methods that stabilize polyphenols in high‐quality food products would be highly desirable for consumers eager to conveniently take advantage of polyphenol health benefits.

Polyphenolic compounds naturally bind to proteins through hydrophobic interactions, hydrogen bonding, and covalent reaction; this affinity results in insoluble protein–polyphenol precipitates (Hagerman, 2012). Typically, this interaction is an undesirable trait in the food industry, for instance the hazing that develops in beverages with high polyphenol content, or the astringent response induced by polyphenols interacting with salivary proteins (Hagerman, 2012). However, these protein–polyphenol interactions have been exploited to develop stable colloidal protein–polyphenol aggregate particles. The particles are utilized as ingredient complexes that bind and concentrate fruit‐ and vegetable‐derived polyphenols to healthy edible protein isolates while excluding excess sugar or water from the polyphenol source and mitigating the astringency typically associated with concentrated flavonoids (Grace et al., 2015). These protein–polyphenol particles have been created with diverse protein and polyphenol sources, including commercially available soy, peanut, whey, rice, pea, and hemp proteins complexed with polyphenols from cranberry, blueberry, muscadine and Concord grapes, cinnamon, green tea, kale, and blackcurrant among others (Grace et al., 2013, 2015; Lila et al., 2017; Plundrich et al., 2014; Roopchand, Kuhn, Krueger et al., 2013; Yousef et al., 2014). The presence of polyphenols improves several food functionality traits including reducing protein reactivity that results in beverage gelling or hardening of bar formulations, stabilizing food product macrostructures such as foams, and improving the stability of polyphenols (Foegeding et al., 2017). These particles, therefore, preserve the fruit bioactives in a convenient, healthy, cost‐effective form that is compatible with many food product formulations.

The complexation conditions, physical characteristics, functional properties, and bioactivity of these particles have been previously examined (Foegeding et al., 2017; Plundrich et al., 2014; Roopchand, Kuhn, Rojo et al., 2013; Schneider et al., 2016); however, the impact of protein isolate characteristics on the particle functionality has yet to be fully investigated. The mechanisms driving polyphenol binding with native protein structures have been well studied; binding is dependent on both polyphenol and protein mass, rigidity or flexibility, chemical properties that promote hydrogen bonding or hydrophobic interactions, and binding stoichiometry (Hagerman, 2012). However, extrapolating these interactions to processed protein isolates would be inaccurate as the protein isolate manufacturing process subjects proteins to many different operations including thermal, enzymatic, and chemical treatments. The resulting protein preparations have structures significantly altered from their native state and do not even resemble their denatured state, but rather have new physicochemical properties of commercial interest such as improved solubility, heat stability, gelling, or emulsifying ability (Barac et al., 2015; Cha et al., 2020; Ma et al., 2018; Wang et al., 2018).

Yellow field pea, like other pulse crops, is a low‐cost, low‐input, sustainable, and high‐quality source of protein that has low environmental footprint. Pea seeds contain approximately 20%–30% protein, the majority of which are globulins (legumin, vicilin, and convicilin) (70%) and albumins (30%) with prolamins and glutelins present as minor components (Barac et al., 2015; Stone et al., 2015). Pea protein isolate is considered an attractive alternative to soy due to comparable nutritional and functional characteristics, as well as being less allergenic (Barac et al., 2015; Stone et al., 2015). Several manufacturers now offer pea protein isolates marketed with various protein contents, purities, and functional properties of commercial interest. Protein isolates from the same plant source but derived from different manufacturers and manufacturing processes are hypothesized to produce protein–polyphenol particles with distinctive physical, nutritional, and bioactive properties.

The overall aim of this study was to explore the functional properties of protein–polyphenol particles prepared with nine commercial pea protein isolates possessing a variety of physicochemical properties of commercial interest. The pea proteins were complexed with increasing amounts of polyphenols extracted from cranberry pomace, and the functional characteristics of the resulting protein–polyphenol particles, including phytochemical content, pH dependence of solubility, and in vitro pepsin and pancreatin digestion rates, were examined. It is important to investigate and optimize these physicochemical properties, as they are vital to the color, texture, sensory quality, and structural and health functionality of the resulting food products.

## 2 MATERIALS AND METHODS

### 2.1 Chemicals and reagents

Folin‐Ciocalteu reagent, gallic acid, pancreatin (porcine), pepsin (porcine), sodium bicarbonate, sodium chloride, and hydrochloric acid were purchased from Sigma‐Aldrich Inc. Cyanidin‐3‐*O*‐glucoside was purchased from ChromaDex. 2,4,6‐trinitrobenzene sulfonic acid (TNBSA reagent) was purchased from Fisher Scientific. Coomassie Plus Reagent and Bovine Serum Albumin standard was purchased from Thermo Scientific. Bio‐Safe Coomassie Stain, Laemmli sample buffer, 4%–20% TGX Precast Protein Gels, Precision Plus Dual Color Protein Standard (10–250 kDa), and 10X Tris‐Glycine‐SDS buffer were purchased from Bio‐Rad Laboratories Inc. 4‐dimethylaminocinnamaldehyde (DMAC) was purchased from TCI. Procyanidin B1 (PAC‐B1) was purchased from Extrasyntheses.

### 2.2 Cranberry polyphenol extract preparation

Cranberry polyphenols extracted from pomace were utilized for all complexation reactions. Dry cranberry pomace (400 g, provided by Ocean Spray Cranberries, Inc.) was ground in a Vitamix blender with 4 L 50% ethanol and incubated for 2 hr at 80°C. Solids were removed by filtration through cheese cloth and Whatman paper #1 after cooling. The extraction solvent was removed by rotary evaporation at 40°C followed by lyophilization. The dry cranberry polyphenol extract was dissolved in water with 0.1% formic acid, and total phenolics were (TP) determined using the Folin‐Ciocalteu reagent (detailed below). The concentrations of total phenolics were expressed as mg/ml gallic acid equivalents.

### 2.3 Cranberry polyphenol–pea protein complexes

Pea protein isolates from the VegOtein line (N, MA, P80, P85 grades) were provided by Axiom Foods Inc. Pea protein isolates from the Nutralys line (S85F, S85M, F85F, F85M, and F85G grades) were provided by Roquette. Each protein isolate was dried by lyophilization prior to complexation to ensure that yields were not impacted by differences in product moisture levels.

The methods for complexation of polyphenols with protein isolates have been previously reported (Grace et al., 2013; Roopchand et al., 2012). Briefly, cranberry polyphenol extract (15 ml), diluted to the appropriate TP concentration (0 mg/ml, 0.5 mg/ml, 1.5 mg/ml, 3 mg/ml, 5 mg/ml, and 8 mg/ml) with 0.1% formic acid, was added to protein isolate (1.5 g) to make a 10% (w/v) solution. The protein and polyphenol extract was mixed by inversion for 1 hr at room temperature and then centrifuged for 20 min at 4,000 rpm. The supernatant was discarded, and the remaining precipitated polyphenol–protein complexes were lyophilized to dry. Three replicates of each protein and polyphenol concentration combination were prepared. Final protein–polyphenol particles were stored at −20°C.

### 2.4 Determination of total phenolics and total proanthocyanidins

To characterize the polyphenols bound to proteins, the polyphenols were extracted as previously described (Grace et al., 2013). Briefly, cranberry polyphenol–pea protein complexes (0.25 g) were extracted in 8 ml of 80% methanol and 1% acetic acid in water. The mixture was vortexed, sonicated for 10 min at 40°C, and centrifuged at 4,000 rpm for 10 min at room temperature. This extraction procedure was repeated two more times, and final volumes adjusted to 25 ml in volumetric flasks. Aliquots were stored at −20°C until analysis.

Total phenolics (TP) of extracts were determined using the Folin‐Ciocalteu reagent and a gallic acid standard using the method described by Singleton (Singleton et al., 1999). Briefly, samples were incubated with Folin–Ciocalteu reagent; sodium carbonate was added, and the samples were allowed to develop color in the dark for 90 min before measuring absorbance at 765 nm. Results were obtained on a SpectraMax M3 plate reader with SoftMax Pro software (Molecular Devices, LLC). A standard curve was prepared from 12.5 to 250 µg/ml gallic acid in water. Samples, standards, and controls were analyzed in quadruplicates in 96‐well plates. Control samples (one per plate, *n* = 13) had CV of 3%. Concentrations were expressed as mg gallic acid equivalents per gram dry weight of protein–polyphenol particles.

Total proanthocyanidin (PAC) concentrations of extracts were determined colorimetrically using DMAC and a PAC‐B1 standard as previously described (Prior et al., 2010). Briefly, DMAC was added to samples and absorbance was immediately monitored at 640 nm for 30 min; the maximum value was recorded. Results were obtained on a SpectraMax M3 plate reader with SoftMax Pro software (Molecular Devices, LLC). A standard curve was prepared from 3.125 to 200 µg/ml PAC‐B1 in 75% ethanol 5% HCl. Samples, standards, and controls were analyzed in quadruplicates in 96‐well plates. Control samples (one per plate, *n* = 8) had a CV of 6%. Concentrations were expressed as mg PAC‐B1 per gram dry weight of protein–polyphenol particles.

### 2.5 HPLC anthocyanin and proanthocyanidin analysis

Extracts were filtered through 0.2 µm PTFE filters prior to HPLC analysis of anthocyanins and proanthocyanidins. HPLC analysis of anthocyanins was performed using an Agilent 1260 Infinity system (Agilent Technology Inc.) equipped with a diode array detector and an autosampler maintained at 4°C. Anthocyanins were separated with a Supelcosil LC‐18 column, 4.6 × 250 mm with 5 µm particle size fitted with the appropriate guard (Sigma‐Aldrich). A binary gradient was utilized with solvent A: 5% formic acid in water and solvent B: methanol. The gradient was 10%–20% B over 0–5 min, 20%–25% B over 5–20 min, 25%–35% B over 20–30 min, 35%–90% B over 30–43 min and held at 90% B for 3 min before returning to 10% B over 46–50 min. The column temperature was set to 30°C, and a 1 ml/min flow rate was used. Anthocyanins were characterized by their absorbance at 520 nm and were identified by their retention times and comparison to previous analyses (Grace et al., 2014). Anthocyanins were quantified as mg cyanidin 3‐*O*‐glucoside equivalents per gram dry weight of protein–polyphenol particles.

HPLC analysis of proanthocyanidins was performed using an Agilent 1200 Infinity system (Agilent Technology Inc.) equipped with a fluorescence detector, a diode array detector, and an autosampler maintained at 4°C. Proanthocyanidins were separated with a Develosil 100‐Diol column, 4.6 × 250 mm with 5 µm particle size fitted with the appropriate guard (Phenomenex). A binary gradient was utilized with solvent A: 2% acetic acid in acetonitrile and solvent B: 3% water, 2% acetic acid in methanol. The gradient was 0%–40% B over 0–35 min, 40%–100% B over 35–40 min, and held at 100% B for 5 min before returning to 0% B over 45–50 min and re‐equilibrating at 0% B for 5 min. The column temperature was set to 35°C, and a 0.8 ml/min flow rate was used. Proanthocyanidins were characterized by exciting at 230 nm and recording emission at 321 nm, and were identified by their retention times and comparison to previous analyses (Grace et al., 2014). Procyanthocyanidins were quantified as mg PAC‐B1 equivalents per g dry weight of protein–polyphenol particles.

### 2.6 Protein digestibility

Protein–polyphenol particle digestibility was assessed using simulated gastric and intestinal conditions (Hur et al., 2011). For the gastric phase, 3 mg protein–polyphenol particle was mixed with 1.8 ml of 0.75% saline, 20 mM HCl, and 0.42 U of pepsin. For the intestinal phase, 3 mg protein–polyphenol particle was mixed with 1.8 ml of 0.75% saline, 20 mM sodium bicarbonate buffer, pH 8, and 3 µg pancreatin. Each solution was incubated at 37°C for 3 hr (pepsin) or 4 hr (pancreatin). Aliquots were removed hourly for amine quantification (detailed below). The rates of digestion (µM alanine equivalents produced per hour) were compared. A control sample, consisting of a mixture of all unmodified proteins, was assessed for digestibility on each plate. The control sample digestion rate had an average CV of 6% over all experiments.

The increase in free amino groups in the supernatant due to digestion was measured using 2,4,6‐trinitrobenzene sulfonic acid (TNBSA) utilizing the manufacturers recommendations, slightly modified. Briefly, aliquots of digest reactions (25 µl) were added to 10 µl 1 M sodium bicarbonate, pH 8, to quench the digestion reaction. TNBSA solution (50 µl of 0.01% TNBSA solution in 100 µM sodium bicarbonate, pH 8) was added, and the reaction was allowed to incubate at 37°C for 2 hr before quenching with 35 µl of 1 M HCl. The absorbance of the solution was measured at 335 nm. Results were obtained on a SpectraMax M3 plate reader with SoftMax Pro software (Molecular Devices Molecular Devices, LLC). A standard curve of alanine was prepared from 0 to 1,000 µM. Samples, standards, and controls were analyzed in triplicate in 96‐well plates.

### 2.7 SDS‐PAGE analysis

Protein–polyphenol particles and unmodified pea protein isolate were visualized by SDS‐PAGE using a Bio‐Rad Mini‐PROTEAN Tetra Cell (Bio Rad) with 4%–20% Mini‐PROTEAN TGX precast polyacrylamide gels (8.6 × 6.7 cm) under non‐reducing conditions. A 1% solution (w/v) of samples at pH 8 was further solubilized in 1% SDS and 4X Laemmli sample buffer. Samples were denatured at 75°C for 10 min and centrifuged for 5 min at 15,000 rpm. Gels were run at constant current of 45 mA for 100 min at room temperature in premixed Tris‐Glycine‐SDS electrophoresis buffer, containing 25 mM Tris, 192 mM glycine, 0.1% SDS, at pH 8.3. Gels were stained using Bio‐Safe Coomassie Premixed Stain and imaged with ChemiDoc MP Imaging System hardware and software (Bio Rad). Molecular weights were estimated using Bio‐Rad Precision Plus dual color protein standard from 10 to 250 kDa.

### 2.8 Solubility

Protein solubility was analyzed as previously described (Deng et al., 2019) with some modification. Briefly, 1% solutions of protein–polyphenol particles were pH adjusted to 2, 3, 4, 5, 6, 7, 8, 9, or 10 with 0.1 M HCl or 0.1 M NaOH. Samples were mixed by inversion and were allowed to incubate overnight before centrifugation for 10 min at 4,000 rpm. Soluble protein was quantified using the Bradford method following the manufacturers protocol. Briefly, 240 µl Coomassie Plus reagent was added to 8 µl sample and absorbance measured at 595 nm within 5 min. Results were obtained on a SpectraMax M3 plate reader with SoftMax Pro software (Molecular Devices Molecular Devices, LLC). A standard curve of bovine serum albumin was prepared from 50 to 1,000 µg/ml in either 0.1% formic acid in water for acidic samples, water for neutral samples, or 20 µM sodium bicarbonate (pH 9) for basic samples. Samples, standards, and controls were analyzed in triplicates in 96‐well plates. Control samples (one per plate, *n* = 18) had a CV of 9%. Soluble protein concentrations were expressed as µg/ml BSA.

### 2.9 Statistics

Statistical analyses were performed using Prism 6.07 (GraphPad Software). Data were analyzed by nonparametric one‐way ANOVA, and analyses of differences between experimental groups were made using Tukey's multiple‐comparison tests with a significance threshold of 0.05.

## 3 RESULTS AND DISCUSSION

### 3.1 Cranberry polyphenol extract analysis

**FIGURE 1:**
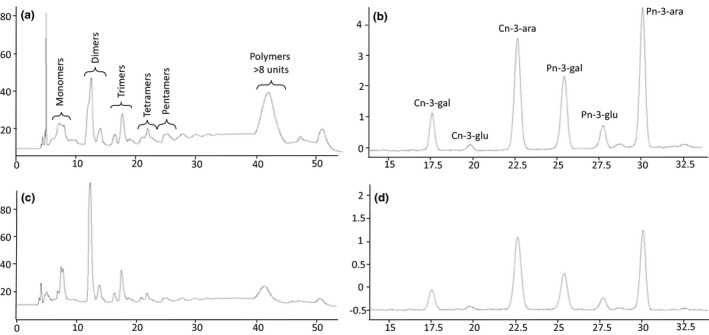
HPLC profiles of proanthocyanidins (a) and anthocyanins (b) from cranberry pomace extract, and representative HPLC profiles of proanthocyanidins (c) and anthocyanins (d) extracted from protein–polyphenol particles

Cranberry pomace, consisting of cranberry seeds, skins, and stems, is a by‐product of juice processing and is a rich source of phytoactive compounds. Substantial amounts of proanthocyanidins, anthocyanins, flavonoids, and phenolic acids can be removed from pomace by food‐compatible extraction (Roopchand, Krueger et al., 2013). The cranberry pomace extract (CPE) prepared in this study was examined by HPLC and compared to both cranberry juice and cranberry pomace extracts previously described (Grace et al., 2014; Roopchand, Krueger et al., 2013; White et al., 2010). As expected, the CPE contained diverse proanthocyanidins; monomers, dimers, trimers and higher oligomers of both A‐type and B‐type linkages were detected (Figure 1a). Additionally, six anthocyanin glycosides—arabinose, galactose, and glucose derivatives of both cyanidin and peonidin—were detected in the CPE (Figure 1b).

### 3.2 Pea protein isolate

In this study, nine pea protein isolate products from two manufacturers (Roquette and Axiom Foods Inc.) were selected for the current study (Table S1). Both manufacturers report using a water extraction process and describe their products as having excellent emulsifying and gelling properties as well as having high water and fat retention capacities. Nutralys pea protein isolates (Roquette) report at least 83% protein content in their isolates and provided base grade (F grades), as well as improved solubility grade (S grades) in particle sizes of fine (F), medium (M), and large (G) (F‐grade only) for the current study (https://www.roquette.com/featured‐ingredient‐food‐vegetal‐proteins‐nutralys‐pea, personal communication). VegOtein pea protein isolates (Axiom Foods Inc.) provided a base‐grade isolate product (P grade) with 80% protein content for the current study as well as additional derivative products including protein enriched (P85 grade), chemically altered products such as the partially hydrolyzed (MA grade), and neutralized (N grade) in which aromatic compounds are removed by oxidative processes (http://axiomfoods.com/protein‐solutions, personal communication). This physically (particle size) and chemically (hydrolyzed and oxidized) diverse protein collection allows investigation into their effects on physicochemical properties of the protein–polyphenol particles.

### 3.3 Complexation & polyphenol analysis

**FIGURE 2:**
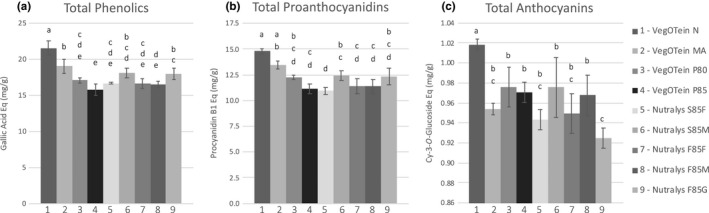
Total phenolics (a), total proanthocyanidins (b), total anthocyanins (c) extracted from nine pea protein isolates enriched with 8 mg/ml cranberry polyphenols. Significance calculated using Tukey's post hoc test where different letters represent statistical differences with *p* < .05 or less

The nine pea protein isolates were complexed with CPE at six different polyphenol concentrations including a non‐polyphenol control. The total phenolics (TP) content of the CPE was determined by Folin‐Ciocalteu assay and diluted to the desired TP concentrations: 8 mg/ml, 5 mg/ml, 3 mg/ml, 1.5 mg/ml, 0.5 mg/ml, and 0 mg/ml gallic acid equivalents. Briefly, the protein–polyphenol particles were made by mixing pea protein isolate and CPE (10% w/v) for 1 hr, and the particles were separated by centrifugation and dried by lyophilization. Each complexation reaction was repeated in triplicate resulting in 162 total samples. Bound polyphenols were extracted by acidified methanol; the TP and total proanthocyanidins (PACs) were determined, and the phytochemical profiles of the extracts were analyzed by HPLC (Figures 1 and 2).

All nine proteins isolates were able to bind polyphenols from CPE, and the HPLC profiles of the extracted polyphenols were similar to the CPE starting material. A representative profile of an extract from a protein–polyphenol particle prepared with 8 mg/ml CPE is presented in Figure 1c,d indicating that the complexation reaction was not selective and proteins were able to capture the heterogeneous mix of polyphenols present in the CPE. As expected, the amount of polyphenols bound by the proteins was proportional to the concentration of polyphenols in the complexation reaction (Figure S1) (Grace et al., 2013; Roopchand et al., 2012). In this study, the protein isolates were not saturated with polyphenols, and the data suggest that the proteins have binding capacity for more polyphenols. 8 mg/ml CPE, however, is the highest reported complexation concentration in literature, and other studies have utilized cranberry polyphenols with TP equivalents of 5 mg/ml and 2 mg/ml (Grace et al., 2013; Schneider et al., 2016), suggesting that achieving full saturation of proteins with cranberry polyphenols is limited by the solubility and concentration of the polyphenols in the complexation reaction rather than the protein binding capacity.

While the protein isolates were all able to bind polyphenols to some degree, they differed significantly in their binding capacity (Figure 2). The particles made from Nutralys protein isolate, which differ mainly in particle size, showed few significant differences in polyphenol binding, whereas many significant differences were observed among the more chemically diverse VegOtein‐derived particles (Figure 2; Table S1). VegOtein N, for instance, complexed 12%–27% more cranberry phenolics than any other pea protein isolate (Figure 2a). The removal of aromatic compounds and increase in oxidation during production of VegOtein *N* (Table S1) may have resulted in improved hydrophobic and hydrogen bonding of polyphenols to the protein resulting in the higher binding capacity observed. Previous work has shown that proteins from different plant sources have differing capacity to sorb cranberry polyphenols (Grace et al., 2013); the current work shows that proteins from the same plant source also have differing capacities to bind polyphenols depending on their manufacturing processes. Some processes have little effect on polyphenol binding, such as changes in particle size within the same grade or changes in protein concentration within the same grade (Figure 2a). Other manufacturing processes, such as the oxidative treatments applied to VegOtein N, significantly alter polyphenol binding behavior.

Pea proteins complexed with the highest concentration of cranberry polyphenols (8 mg/ml TP) were able to bind 15.8–21.6 mg/g TP (gallic acid equivalents), 10.9–14.8 mg/g total PACs (PAC‐B1 equivalents), and 0.92–1.02 mg/g total anthocyanins (cyanidin 3‐*O*‐glucoside equivalents) (Figure 2). Previous work testing the capacity of protein‐rich products to sorb cranberry polyphenols found that pea protein isolate bound 21 mg/g TP, 15 mg/g PACs, and 2.5 mg/g anthocyanins which is within the same range as our results (Grace et al., 2013). Commercial fresh cranberries contain 350 mg TP, 133 mg PACs, and 76 mg total anthocyanins per 100 g fresh weight (Grace et al., 2014). For the protein–polyphenol particles prepared in this work to reach the equivalents found in 100 g fresh cranberries, 16 g particles would achieve equivalent TP, 9 g particles for equivalent PACs, or 76 g for equivalent anthocyanins. It is likely that the comparatively high equivalence required for anthocyanins is due to their decreased presence in the pomace starting material; a 4.6‐fold decrease in extractable anthocyanins for pomace has been reported (White et al., 2010). Anthocyanins may be less abundant in the tissue types that make up pomace, or it is possible that anthocyanins are a more labile polyphenol class that is degraded during preparation of the pomace.

**FIGURE 3:**
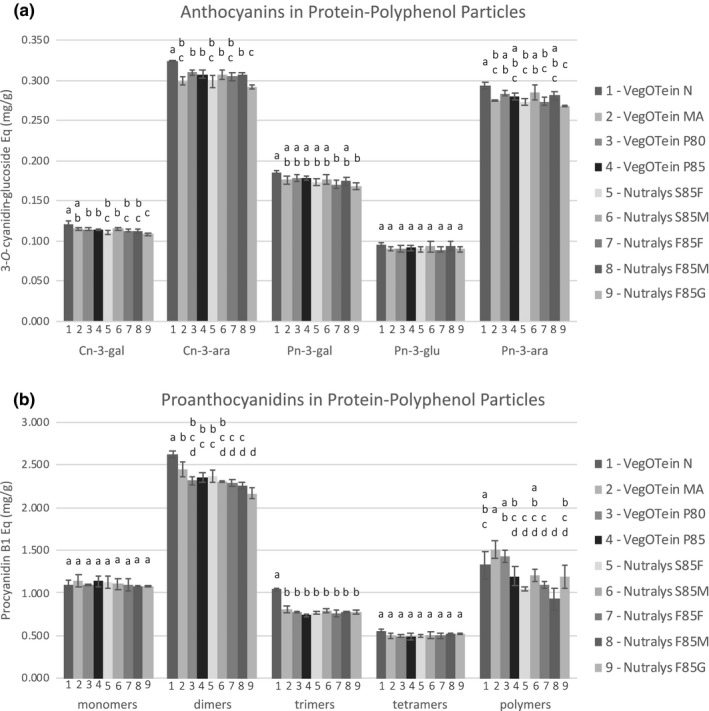
Differences in individual anthocyanin (a) and proanthocyanidin (b) species extracted from nine pea protein isolates enriched with 8 mg/ml cranberry polyphenols. Significance calculated using Tukey's post hoc test where different letters represent statistical differences with *p* < .05 or less

Individual species of PACs and anthocyanins (Figure 3) extracted from protein–polyphenols particles prepared with 8 mg/ml CPE were examined by HPLC. VegOtein N shows the highest amount of dimer, trimer, and tetramer PACs (Figure 3a), mirroring the results from the total PAC assay (Figure 1b). A high degree of variation was observed between higher‐order polymer PAC (degree of polymerization >8) species bound to the proteins (Figure 3b). The affinity of polymeric PACs for proteins is highly dependent on the protein size, conformation, and charge (Hagerman, 2012), so the binding variation observed here may be reflective of the heterogeneity of the underlying protein structures in this collection. Little variation was observed in the binding of individual anthocyanin species between the nine proteins (Figure 3b). Only VegOTein N showed 4%–9% more anthocyanin binding more than the other pea protein isolates. This lack of variation is likely due to lower respective content of anthocyanins in CPE, and it is expected that the functionally different pea proteins will also have diverse anthocyanin binding properties. Further experiments using a polyphenol source with a higher concentration of anthocyanins would be needed to examine this hypothesis.

No significant difference in TP between the control particles, prepared with 0 mg/ml CPE, and the particles prepared with 0.5 mg/ml CPE was detected; increases in TP attributable to cranberry polyphenols were observed for particles complexed with 1.5 mg/ml CPE and above (Figure 2a). Protein–polyphenol particles prepared with low polyphenol content CPE (0.5 and 1.5 mg/ml) are not able to concentrate enough bioactives for interest as a commercial food ingredient and reveal few significant differences between the pea protein isolates. Therefore, further functional assays were carried out with particles complexed with 3 mg/ml, 5 mg/ml, and 8 mg/ml CPE.

### 3.4 Protein–polyphenol particle digestibility

**FIGURE 4:**
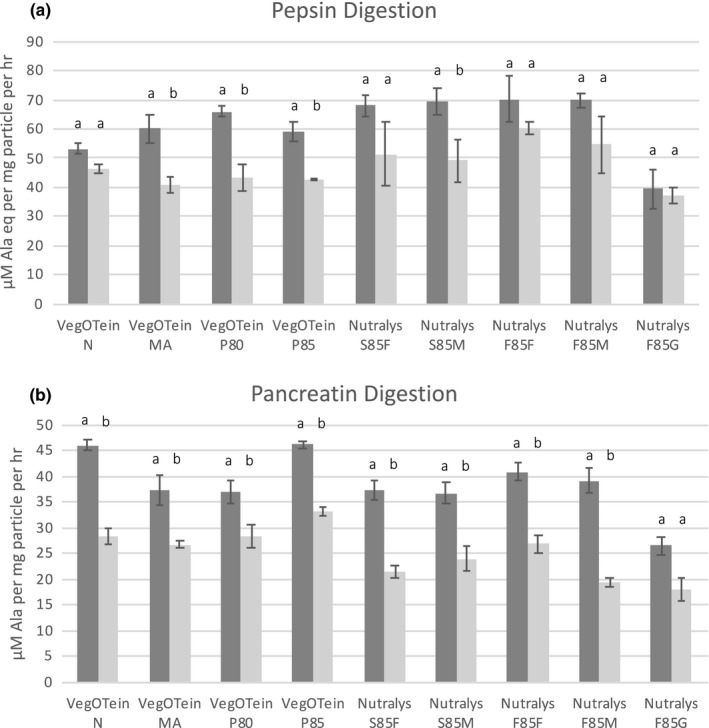
Rates of pepsin (a) and pancreatin (b) digestion of nine pea protein isolates enriched with 8 mg/ml of cranberry polyphenols (light gray) compared to 0 mg/ml control particles (dark gray). The increase in amines (alanine equivalents) were measured over 3 hr (pepsin) or 4 hr (pancreatin). Significance calculated using Tukey's post hoc test where different letters represent statistical differences with *p* < .05 or less

Protein digestibility was examined by subjecting the protein–polyphenol particles to pepsin digestion in acidic conditions to mimic gastric digestibility, as well as pancreatin digestion in basic conditions to simulate intestinal digestion. Complexation with polyphenols slowed the digestion rate of the proteins, the effect becoming more pronounced with increasing polyphenol concentration (Figure 4; Figure S2). Digestion rates for particles complexed with 8 mg/ml CPE decreased an average of 25% for pepsin and 35% for pancreatin compared to control particles complexed with 0 mg/ml CPE. Protein particle size seemed to play an important role in digestibility; protein–polyphenol particles prepared with Nutralys F85F and S85F (the protein isolates with small particle size, Table S1) displayed a 9%–38% higher rate of pepsin digestion (Figure S3) whereas particles prepared with Nutralys F85G (the largest particle size) had the lowest digestion rate for both control and polyphenol complexed particles. Similarly, other studies show increased particle size resulted in decreased digestibility of protein isolate subjected to different ball milling treatments (Wang et al., 2018).

Different amino acid sequences and structures can impart a wide range of protein digestion rates. Fast digesting and slow digesting proteins both increase satiety but by different mechanisms, for instance by quickly increasing plasma amino acid concentration or by delaying gastric emptying (Foegeding et al., 2017). Human clinical trials have demonstrated that polyphenols also increase satiety due in part to their well‐documented inhibition of digestive proteases (Martinez‐Gonzalez et al., 2017). The impact of polyphenols that are protein‐bound on digestive enzymes and proteolysis of the protein component is less well understood and disputed in the literature. Some studies show enhanced digestibility of the protein component (Plundrich et al., 2015; Shen et al., 2014), and others show reduced digestibility upon polyphenol binding (Stojadinovic et al., 2013; Świeca et al., 2013). Structural studies, however, agree that polyphenol‐mediated stabilization or destabilization of the protein structure results in decreased or increased digestibility respectively, and this protein specific effect can be dependent on pH or polyphenol binding affinity (Shen et al., 2014; Stojadinovic et al., 2013). The decrease in both pepsin and pancreatin digestion rate observed for the protein–polyphenol particles in this work suggests that the cranberry polyphenols have a stabilizing effect on the pea proteins that is not dependent on pH (Figure 4). Pea proteins are considered highly digestible, so a decrease in the digestion rate of this protein does not imply a decrease in the nutritional quality of the protein as satiety could be increased by other mechanisms (Rutherfurd et al., 2015).

Previous shelf stability and in vitro digestion experiments have shown that the polyphenol component of these particles is highly protected from degradation and more bioaccessible than non‐protein‐bound formulations (Correia et al., 2017; Grace et al., 2013; Green et al., 2007; Ribnicky et al., 2014). Improved polyphenol bioefficacy and bioavailability, as measured by an increase in circulating polyphenolic metabolites, are also observed in both animal and human clinical trials with polyphenol–protein particles (Nieman et al., 2013; Roopchand, Kuhn, Krueger et al., 2013; Roopchand, Kuhn, Rojo et al., 2013). Ample evidence now establishes that polyphenols are minimally absorbed in the small intestine, but rather are catabolized by intestinal microbiota in the lower gastrointestinal tract (Williamson et al., 2018). This suggests that the lowered digestion rate of protein component will protect the polyphenols during transit through the gastrointestinal tract and result in improved bioavailability; however, further studies are needed to examine this hypothesis.

This study demonstrates that while the rates of digestion of protein–polyphenol particles are impacted by the presence of polyphenols, they are highly dependent on the nature of the protein starting material. In this study, physical differences between the protein starting material, such as their particle size, had a significant effect on particle digestibility, whereas proteins subjected to chemical processing (Table S1) resulted in fewer digestibility differences in the protein–polyphenol particles.

### 3.5 Protein–polyphenol particle solubility

**FIGURE 5:**
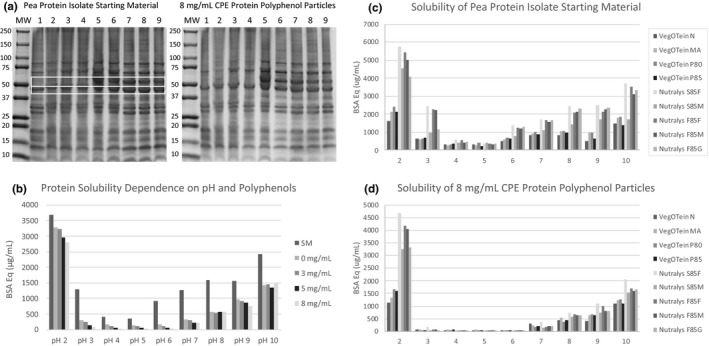
Solubility of pea protein isolates and protein–polyphenol particles. (a) Gradient SDS‐PAGE analysis of nine pea protein isolates starting materials (left) and protein–polyphenol particles prepared with 8 mg/ml cranberry polyphenol extract (right). Lanes (1) VegOtein N, (2) VegOtein MA, (3) VegOtein P80, (4) VegOtein P85, (5) Nutralys S85F, (6) Nutralys S85M, (7) Nutralys F85F, (8) Nutralys F85M, (9) Nutralys F85G. (b) Average solubility of pea protein isolates and protein–polyphenol particles prepared with cranberry polyphenols from pH 2 to pH 10. (c) Solubility of individual pea protein isolate starting materials from pH 2 to pH 10. (d) Solubility of individual protein–polyphenol particles prepared with 8 mg/ml cranberry polyphenol extract

The solubility of the protein–polyphenol particles was investigated by SDS‐PAGE analysis as well as by measuring the soluble protein content of 1% (w/v) pooled preparations at a range of acidic and basic pH levels (Figure 5). The pea protein starting material, control particles prepared with 0 mg/ml CPE, and protein–polyphenol particles prepared with 3, 5, and 8 mg/ml CPE all showed typical protein solubility behavior in the tested pH range with very low solubility near the isoelectric point (pH 4–5), and higher solubility at the pH extremes (pH 2, 10) near the pK_a_ of acid and amine groups respectively (Figure 5). SDS‐PAGE analysis of soluble proteins from pea protein isolate starting materials from different manufactures shows similar protein compositions reflecting their common plant source (Figure 5a). Pea protein isolates within the same manufacturing lines have very similar SDS‐PAGE profiles; however, there are some differences between the Nutralys and VegOtein lines, likely a result of the difference in protein preparation techniques between the manufacturers (Figure 5a). This same observation has been made in related studies; protein extraction method seems to impact the SDS‐PAGE profiles the most, and further protein processing impacts the secondary structure of proteins but the amino acid sequence (primary protein structure) remains the same (Cha et al., 2020; Ma et al., 2018; Wang et al., 2018).

Substantial differences in solubility between pea protein isolate starting materials (SM) were observed; isolates from the same manufacturer had similar solubility performances, but Nutralys isolates were on average 50% more soluble than isolates from the VegOTein line (Figure 5c). These differences in solubility could be explained by the differences observed by SDS‐PAGE analysis between starting materials. For instance, a band around 60 KDa (indicated in the top white box in Figure 5a) which likely corresponds to legumin subunits is present in Nutralys samples (lanes 5–9) but not in VegOTein samples (Lanes 1–4). These legumin subunits, which are comprised of covalently linked acidic and basic polypeptide chains, can form monomers, dimers, and hexamers depending on the ionic or pH environment and may contribute to the differences in solubility observed between the samples (Barac et al., 2015). Additionally, Nutralys samples have higher percentage of the protein in the 45 kDa region (indicated by the bottom white box in Figure 5a), which likely corresponds to a vicilin subunit. Vicilin is a protein with a more hydrophilic surface than other pea proteins that could explain the increased solubility of Nutralys starting materials (Figure 5c) (Stone et al., 2015). Many different extraction techniques can be used to produce protein‐rich products, and previous experiments have shown that the choice of extraction technique (for example, alkaline extraction–isoelectric precipitation, salt extraction‐dialysis or micellar precipitation) produces protein products of significantly different composition and physiochemical properties (Stone et al., 2015). Even slight differences in protein isolate processing, such as adjusting the amplitude or temperature in ultrasound treatments or different pH during isoelectric purification, can result in solubility differences among related protein products (Cha et al., 2020; Ma et al., 2018). Our data also suggest that the different methods of extraction employed by the manufacturers have the largest impact on solubility—resulting in the observed differences between VegOTein and Nutralys samples—and that further processing and modifications to generate the different grades (for example, the N, MA, P80 and P85 grades within the VegOTein line) have less significant impacts.

The protein–polyphenol complexation procedure, which relies on precipitation of proteins, significantly reduces particle solubility. Control particles (prepared with 0 mg/ml CPE) averaged a 56% decrease in solubility compared to the unmodified protein starting material (Figure 5b). Increased polyphenol loading results in a further decrease in solubility; however, the magnitude of this effect was dependent on pH (Figure 5b). At mid‐range pHs (pH 3–6), the particles prepared with 8 mg/ml CPE experienced a 75% decrease in solubility compared to control particles, while at basic pHs only a 14% decrease in solubility due to polyphenol binding was observed. This decrease in solubility due to polyphenol addition was not unexpected, as previous work has repeatedly demonstrated similar decreases in protein solubility after interaction with polyphenols (Correia et al., 2017; Ozdal et al., 2013; Rawel et al., 2002). Interestingly, the same manufacturer‐dependent differences in solubility observed for the protein starting materials were not observed for the resulting polyphenol particles (Figure 5c,d; Figure S4). Except at very low pH, particles derived from both Nutralys and VegOTein protein isolates had similar (lower) solubilities (Figure 5c,d). This indicates that the solubility of the protein–polyphenol particle is at least somewhat independent of the solubility of the protein isolate starting material. Previous work has indicated that the solubility decrease can be dependent on a number of factors, including polyphenol size and chemical structure, and protein amino acid sequence and structure (Correia et al., 2017; Ozdal et al., 2013; Rawel et al., 2002). Even the method used to dry the protein–polyphenol particles can influence solubility, and in one study, spray drying was found to produce more soluble particles than oven drying or freeze drying (Correia et al., 2017). In this study, although the manufacturing procedures led to differences in solubility for the protein starting materials, the final protein–polyphenol particles were derived from the same plant protein source and resulted in the similarly depressed solubilities (Figure 5).

Protein ingredient solubility is an essential functional characteristic that influences many physical properties including foaming capacity and stability, water holding, gelling, viscosity, oil holding, and emulsion capacity and stability properties in food (Deng et al., 2019; Stone et al., 2015). These physical properties, in turn, are vital to the color, texture, and sensory quality of food products. The decrease in solubility observed for protein–polyphenol particles may adversely affect these characteristics, although others have suggested that the polyphenols can play a stabilizing role in these food structures (Foegeding et al., 2017). Further experiments are needed to fully explore the impact of polyphenols on these functional properties.

## 4 CONCLUSION

In this study, several pea protein isolate starting materials with different physical and chemical properties were utilized to prepare protein–polyphenol particles with cranberry polyphenols. The VegOTein protein isolate line represented more chemical diversity, with hydrolyzed and oxidized products, whereas the Nutralys line had more physical diversity, with numerous particle size differences among the products. Many studies have investigated the effects of processing on the properties of a single protein isolate, but to our knowledge none have investigated the effect of protein processing on the physicochemical properties of an ingredient system such as protein–polyphenol particles. The choice of protein isolate starting material had significant impact on the functional properties of protein–polyphenol particles. In this work, the biggest variations in polyphenol binding were observed among protein samples with chemical rather than physical differences; VegOtein *N* (aromatic compounds removed from base grade leading to some product oxidation) bound the most polyphenols of all isolates tested. Digestibility of particles, however, was more dependent on physical differences of protein starting material; Nutralys F85G (the product with largest particle size in the current study) resulted in the lowest rate of both pancreatin and pepsin digestion. Protein–polyphenol complexes displayed 50%–75% decreases in protein solubility compared to unmodified starting materials and non‐polyphenol control particles; however, this decrease was independent of protein starting material properties and was mainly due to the complexation procedure and polyphenol binding. The desired functional properties of the protein–polyphenol particle food ingredients are considerably influenced by the properties of the protein isolate starting material, and care must be taken to select the starting material with the appropriate physicochemical properties for the desired application.

## CONFLICT OF INTEREST

The authors declare that they do not have any conflict of interest.

## AUTHOR CONTRIBUTION

**Renee Cilliers Strauch:** Conceptualization (equal); Formal analysis (lead); Investigation (lead); Methodology (lead); Writing‐original draft (lead); Writing‐review & editing (equal). **Mary Ann Lila:** Conceptualization (equal); Funding acquisition (lead); Supervision (lead); Writing‐review & editing (equal).

## ETHICAL REVIEW

This study does not involve any human or animal testing.

## Supporting information

Supplementary Material

## Data Availability

Data available within the article or its supplementary materials.
