# Supplementary material for: Pea protein isolate characteristics modulate functional properties of pea protein–cranberry polyphenol particles
Source: Food Sci Nutr. 2021 May 24;9(7):3740–51. doi: 10.1002/fsn3.2335 (PMC8269610; doi:10.1002/fsn3.2335)
Supplement: Supplementary file 1 — Supplementary Material [file FSN3-9-3740-s001.pdf]

Supplemental Table 1: Pea protein isolate starting material manufacturer and properties

Pea Protein Isolate	Manufacturer	Properties
**VegOTein N**	Axiom Foods Inc.	Aromatic compounds removed from P80 to create neutral flavor, oxidation of product likely
**VegOTein MA**	Axiom Foods Inc.	Partially hydrolyzed form of P80. Highest gel strength, water holding capacity, and emulsion stability of VegOTein line
**VegOTein P80**	Axiom Foods Inc.	Base grade. Water separation purification process
**VegOTein P85**	Axiom Foods Inc.	Water separation purification process, higher protein content
**Nutralys S85F**	Roquette	Improved solubility, fine particle size
**Nutralys S85M**	Roquette	Improved solubility, medium particle size
**Nutralys F85F**	Roquette	Base grade, fine particle size
**Nutralys F85M**	Roquette	Base grade, medium particle size
**Nutralys F85G**	Roquette	Base grade, large particle size

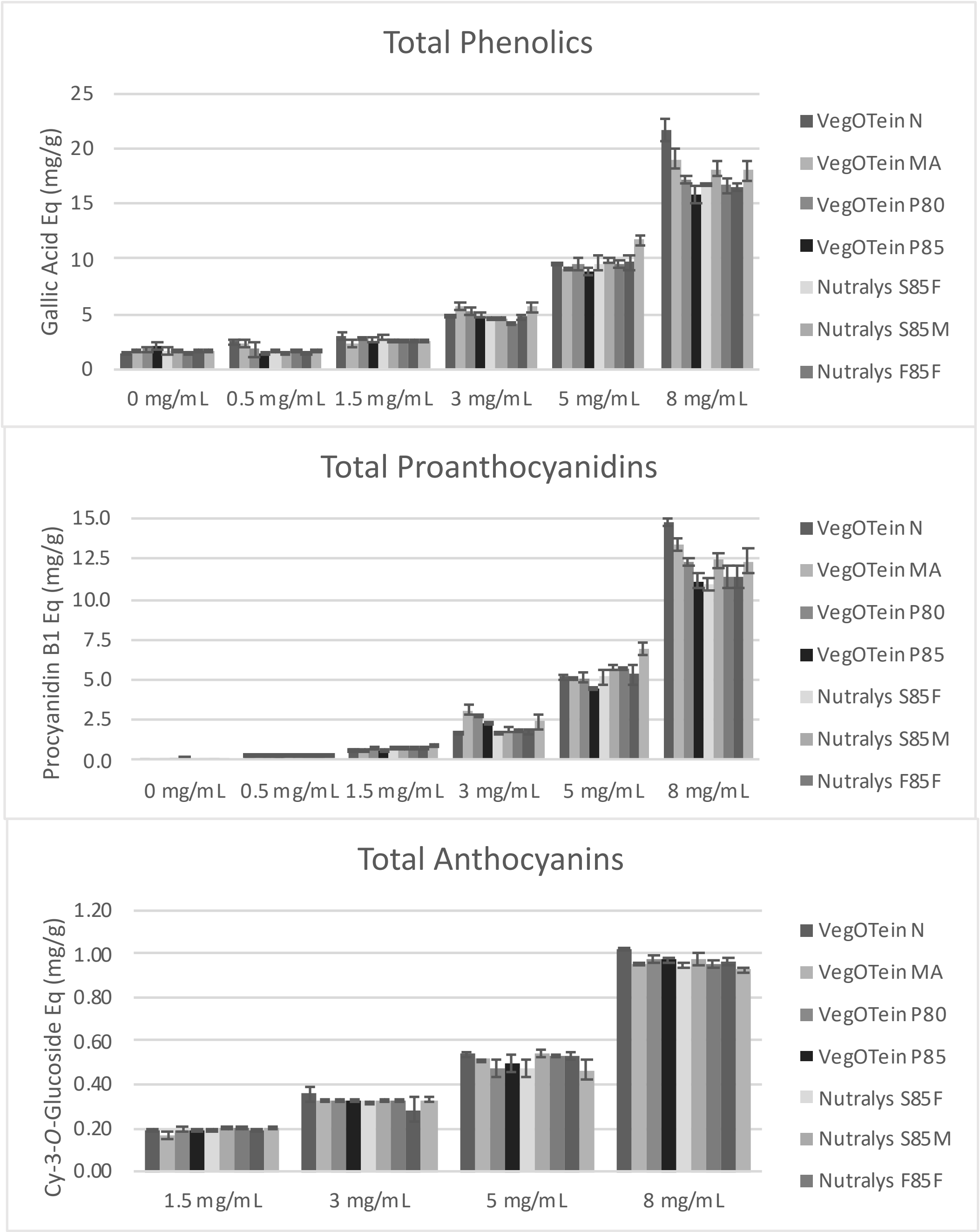

Supplemental Figure 1: Total phenolics (A), total proanthocyanidins (B), total anthocyanins (C) extracted from nine pea protein isolates enriched with different amounts of cranberry polyphenols.

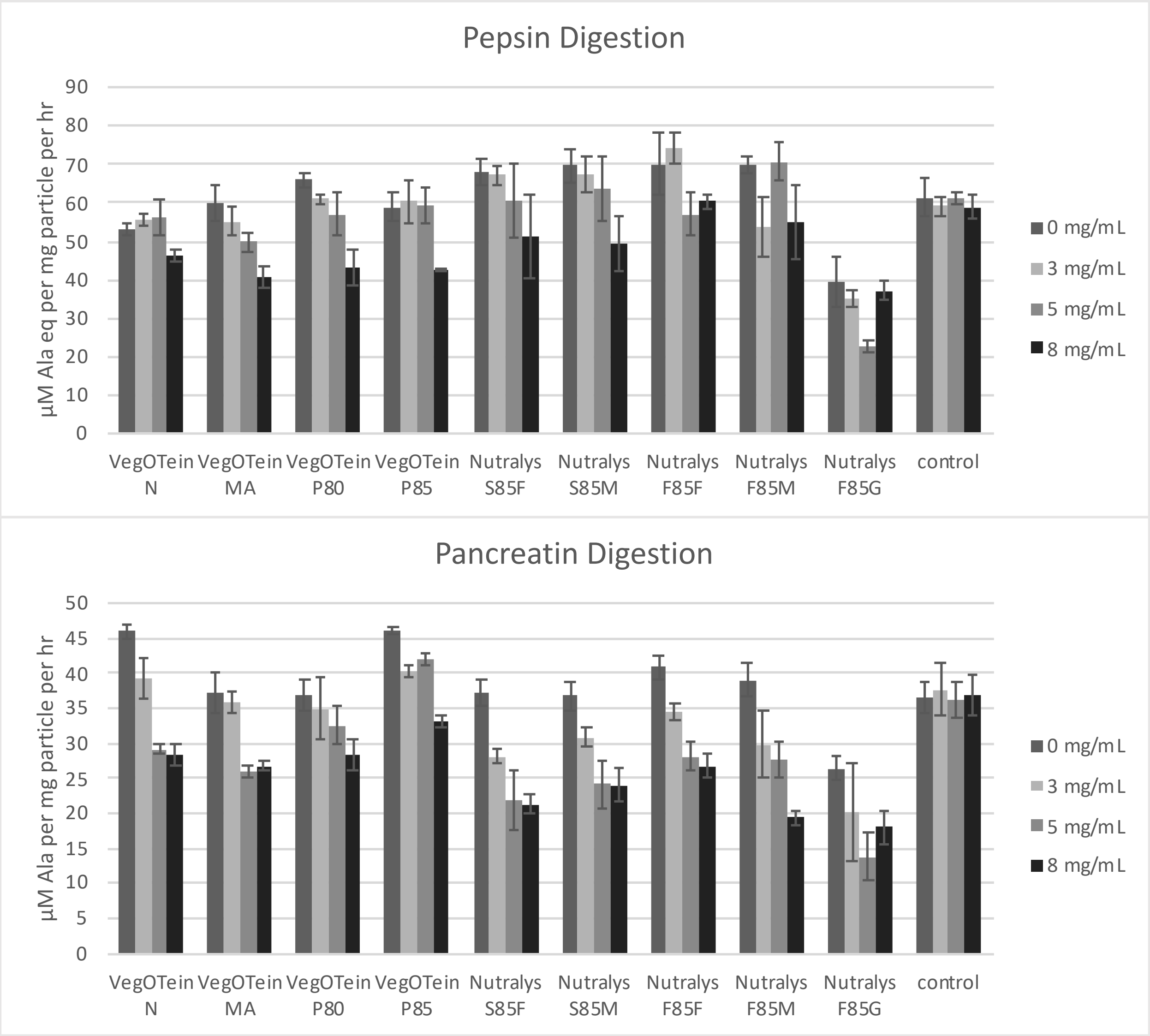

Supplemental Figure 2: Rates of pepsin (A) and pancreatin (B) digestion of nine pea protein isolates enriched with different amounts of cranberry polyphenols. The increase in amines (alanine equivalents) were measured over 3 hr (pepsin) or 4 hr (pancreatin).

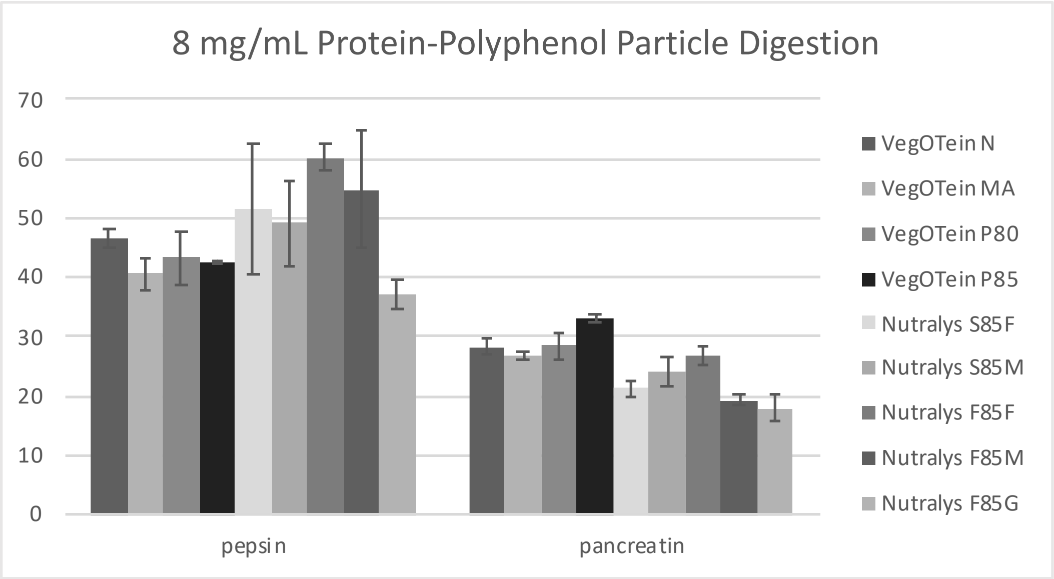

Supplemental Figure 3: Pepsin and pancreatin digestion of nine pea protein isolates enriched with 8 mg/mL cranberry polyphenols.

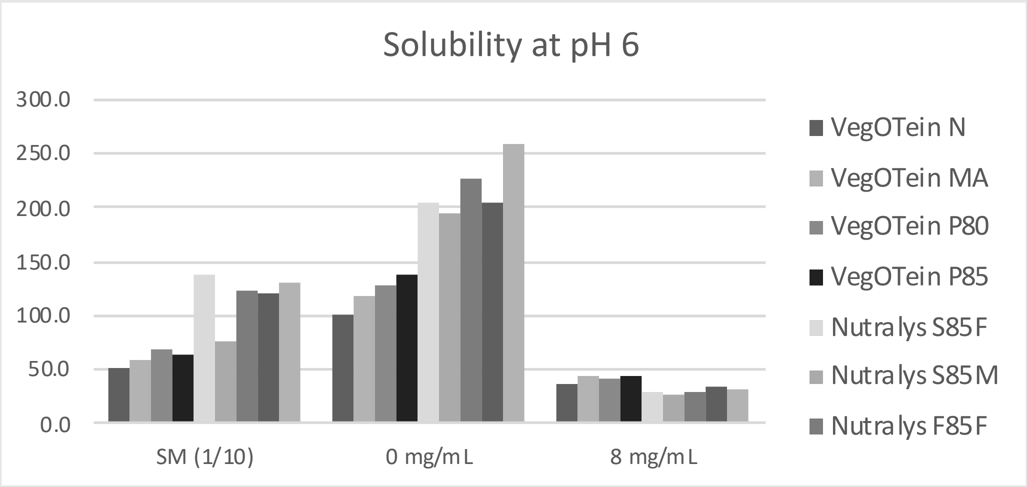

Supplemental Figure 4: Solubility at pH 6 of unmodified protein isolate starting material (10% of solubility for scale), particles prepared with 0 mg/mL CPE, and particles prepared with 8 mg/mL CPE.
